# Beer stein blast to the head a rare case of combined blunt and sharp force trauma

**DOI:** 10.1007/s00414-020-02353-6

**Published:** 2020-07-06

**Authors:** S. N. Kunz, N. Gorges, F. Fischer, J. Adamec

**Affiliations:** 1 Institute of Forensic Medicine, University Hospital Ulm, Prittwitzstr. 6, 89075 Ulm, Germany; 2 Ulm University, Ulm, Germany; 3 Institut für Rechtsmedizin, Ludwig-Maximillians Universität München, Munich, Germany

**Keywords:** Beer stein, Head injury, Assault, Blunt force, Sharp force

## Abstract

Cases of combined blunt and sharp force trauma to the head caused by one striking tool are rare. When beer steins are used as an assault weapon, they can cause blunt traumas upon initial contact phase. If the impact force exceeds the mechanical stability of the beer stein, it breaks into several sharp-edged pieces, which then can cause sharp force trauma injuries due to the interaction between the head and the stein fragments.

We present a case of a 43-year old man, who suffered from blunt and sharp force head traumas due to one single blow with a 1-l beer stein. A forensic-biomechanical analysis of the event, together with witness testimony evaluation and experimental comparison helped to reconstruct the most probable chain of events. Based on these findings as well as on the medical diagnoses and treatment, the assault was assessed as a nonacute life-threatening, but potentially fatal offence. The case was indicted as grievous bodily harm.

## Introduction

The use of blunt objects as an assault weapon with blows launched against the head is a common form of body violence during physical confrontations. Especially in regional restaurants, in larger beer drinking halls or at annual autumn fairs in south Germany and Austria, traditional beer steins made of glass or clay are misused as a striking weapon. In this context, it is not unusual that the beer steins break upon contact with the head, causing blunt- as well as sharp-force injuries. This very special form of a mixture of blunt- and sharp-force trauma resulting from a single blow, mainly aimed at the head, can lead to severe injuries with life-threatening complications. A frequent occurrence of beer stein assault cases is recorded by the police during the annual Octoberfest in Munich, Germany, with numbers ranging from 27 to 53 each year [1–5].

In such cases, it is the task of the forensic medical specialist to determine whether the morphology of the injury is specific enough to draw conclusions about a particular weapon and the manner, in which it was used. A detailed forensic-biomechanical analysis then estimates the injury potential of this particular incident. Together with police investigations, witness testimonies and clinical medical data, a reconstruction of the event with actual and potential injury formation mechanisms is presented to the legal entities.

On the basis of one case report, the present work discusses the main forensic and biomechanical aspects and resulting injuries of 1-l beer stein assaults to the head.

## Case background and clinical reports

### Case background

The victim was a 43-year-old man without known preexisting medical conditions or history. On the day in question, the man visited the annual Munich Octoberfest. He consumed several beers. Afterwards, in a bar nearby, he was involved in a verbal confrontation with two other men. According to his statement, he walked away from the scene, when he suddenly felt a blow to his head and went down to the ground. He did not lose consciousness. According to witnesses, the victim was hit once with a beer stein to the head. The stein broke into several pieces. According to the police report, it was not possible to obtain reliable information considering the striking technique. The police did not secure the fractured pieces of the beer stein.

### Emergency protocol

**Fig. 1:**
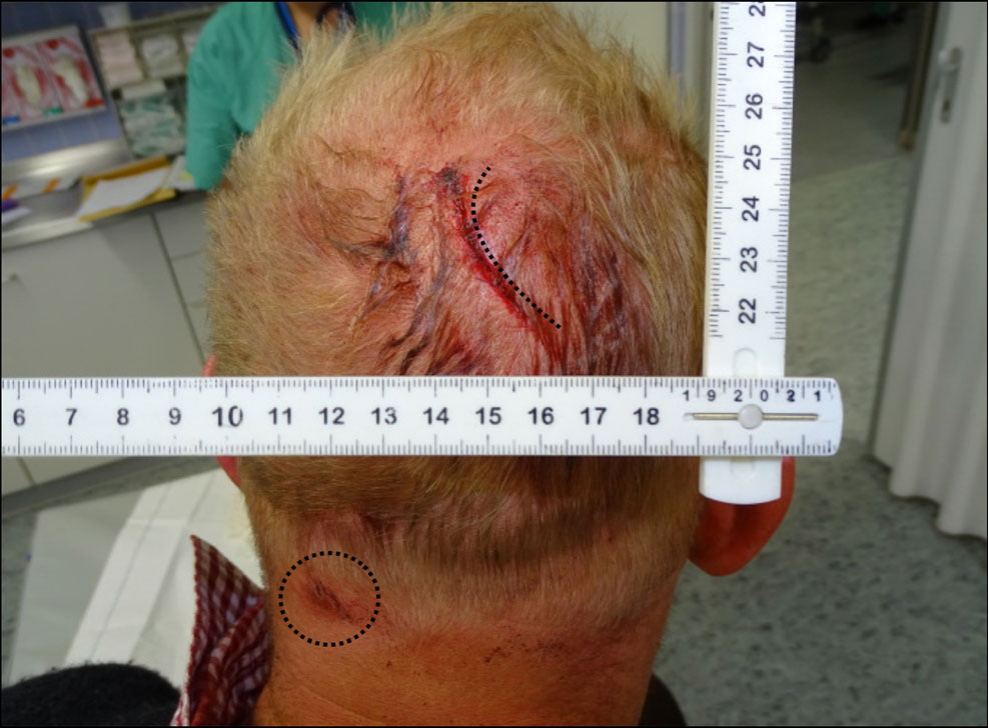
Incised wounds in the right parietal region of the head and on the posterior neck

According to the emergency report, the ambulance arrived on scene shortly after the incident. The man was under the influence of alcohol at that time. He smelled of alcohol and is articulation was blurry. He was conscious and responded to questions accordingly. He suffered one 5-cm long, sharp-edged skin defect in the right parietal region of the head (Fig. 1). In addition, one superficial injury was detectable in the left upper posterior neck region (Fig. 1), measuring approximately 0.8 cm in diameter. Pressure bandages were applied to the wound, and the patient was driven to the hospital.

### CT-scan

**Fig. 2:**
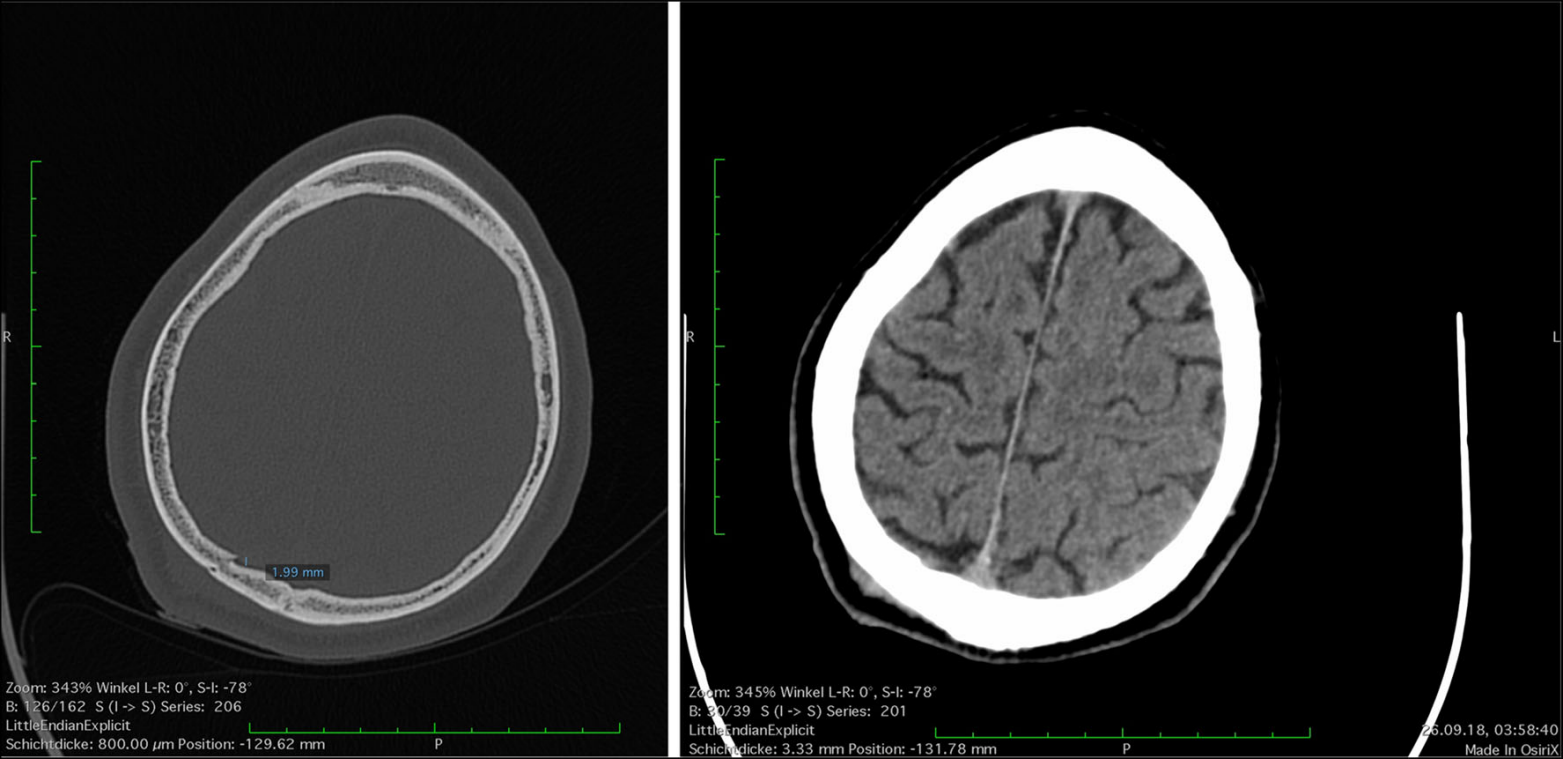
Cranial CT-scan

**Fig. 3:**
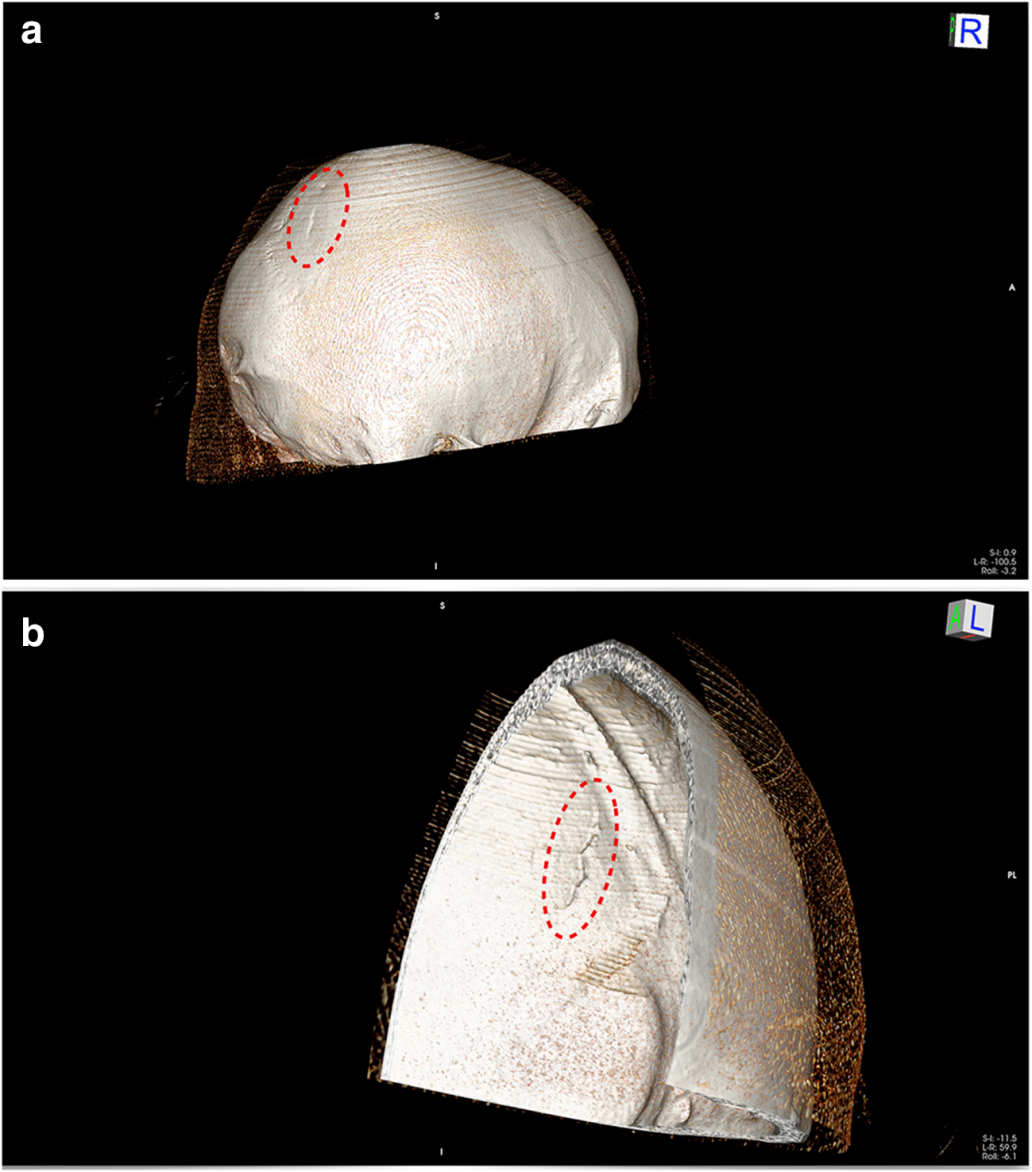
**a** 3D bone reconstruction of the skullcap fracture **b** 3D bone reconstruction of the skullcap fracture

The performed CT-diagnostics revealed an impression fracture (1.99 mm) of the right parietal bone (Figs. 2 and 3a, b), which was accompanied by a haemorrhage to the inner scalp (Fig. 2). A slightly developed subdural haematoma could be identified (Fig. 2). At the fraction site, the parietal bone had a thickness of 6 mm.

### Medical report

**Fig. 4:**
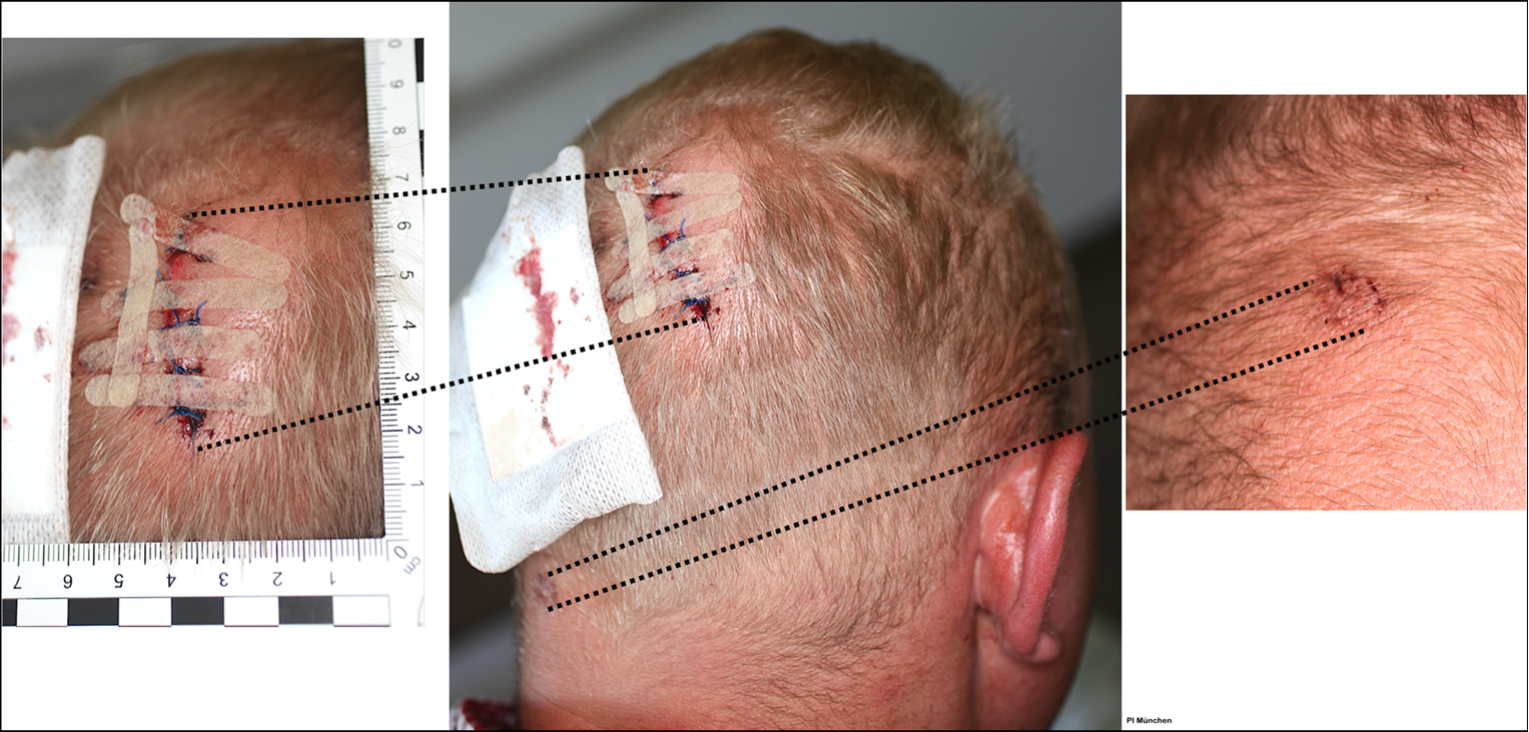
Condition after surgical treatment of the incised wound

The injury in the parietal region of the head was surgically treated with five stitches (Fig. 4). The superficial injury in the neck region did not need surgical treatment. The cranial injury was treated conservatively, and the patient was sent home after 24 h of observation. The victim also showed a fracture of the second metacarpal bone, located within the mid segment of the right hand.

### Toxicological analysis

No toxicological blood analysis was performed, and no breath alcohol analysis was taken.

### Physical examination

At the time of the forensic medical examination of the patient, 9 h after the incident, he already had been treated surgically (Fig. 4). The patient was responsive, cooperative, friendly and outgoing. A body length of 185 cm and a body weight of 90 kg (BMI 26.3) were measured. Apart from the surgically treated, 5-cm long incised wound in the upper right parietal region of the head, a superficial abrasion mark was detectable in the right upper posterior neck region, measuring approximately 0.8 cm in diameter.

## Discussion

Blows with objects to the human head are a very complex phenomenon [6, 7]. From a biomechanical point of view, they are interpreted as an elastic or inelastic collision, depending on resulting injuries to the target or damages to the used object [8]. A blast with a beer stein to the head, as it occurred in the case at hand, is an illustrative example of such an impact. The typical 1-l beer stein weighs approximately 1.3 kg. Independently from the manner in which the beer stein is used as a weapon, it is highly unlikely that its liquid content is still in the container when it strikes a target. Thus, the amount of beer in the stein can be ignored in the context of biomechanical calculations. The traditional Bavarian beer stein is made of glass and has a thickness of approximately 0.6–1.0 cm. The handle is positioned in the midsection of the stein, which makes it an easy-to-handle striking tool. Depending on the relation between the impact tolerance of the skull (here the parietal bone) and the fracture threshold of the stein, severe blunt and sharp force injuries can occur. When the beer stein strikes the head, the impact force is transferred onto the target area until it exceeds the fracture threshold of the beer stein. As soon as the stein breaks, no further blunt skull trauma, but subsequent severe sharp force trauma can occur. Depending on the condition of the beer stein (new vs. used), there can be high variabilities in fracture thresholds. In addition, a high degree of interindividual variability in tissue tolerance can be seen in victims, depending on their physical appearance, age, sex and ethnicity. Thus, beer stein injuries to the head have a complex mechanism of injury formation and need to be evaluated from a forensic-biomechanical point of view.

As documented in Fig. 1, the patient showed a slightly arched injury to the scalp within his parietal region. No additional scratches or abrasion marks were visible in the immediate surroundings of the skin lesion. During the physical examination of the victim, it was not possible to identify any form of soft tissue bridging within the wound or its relatively sharp appearing edges. This was confirmed by the emergency nurse that treated the patient. The basic morphological features of the wound are consistent with sharp trauma. However, there are other factors to be considered in the forensic medical assessment:

- The impression fracture of the skull lies underneath the wound. The fracture morphology is consistent with blunt trauma, since a very fine sharp structure would likely lead to a different fracture pattern.
- The impression fracture proves a high impact force. Such a force can far easier be reached by the whole stein (that might break due to the impact) then by its fragments. Moreover, an impact of the stein leading to its breakup is likely to cause a skin laceration, because a high impact force is acting not only on the stein, but also on the skull and the skin in between.
- Skin lacerations due to impacts with smooth objects can resemble incision wounds.

Taking into account all the information at hand, the assumption seems reasonable that the arched skin wound and the skull fracture resulted from the initial impact of the beer stein on the head.

An additional scratch mark, resulting from a sharp-pointed and at the same time irregularly formed object, was identified in the left upper posterior neck region (Figs. 1 and 4). This neck scratch was caused by contact with a fractured glass piece, a phenomenon, which often occurs in such incidents.

Forensic-biomechanical studies in this specific field could demonstrate that such combined injury pattern is typical for beer stein injuries [9–15]. Based on these studies, a 1-l beer stein, which broke upon impact, was assigned as the used striking weapon. The fact that the shape and number of fractured beer stein pieces was unknown limited a forensic-biomechanical reconstruction. However, based on previous studies [10–15] and taking into account the high fracture threshold of the parietal region of the skull [16–19], a forcefully performed strike to the head with the mechanically robust bottom is likely to have caused the skull fracture at hand. A blast with the side of the beer stein transfers slightly less energy than with the bottom [11]. Thus, the striking technique was reconstructed as a bottom-first strike with high-acceleration in a hammer-like motion.

The fracture of the second metacarpal bone of the right hand was assessed as a fall-related trauma subsequent to the cranial blast with the beer stein.

For the legal assessment of the case, the impact force and the actual and potential injuries are of interest. In a simple biomechanical model that can be used for the impact force calculation, the intensity of the strike can be described by means of the impact velocity and the mass of the stein (the effective mass might be altered depending on the grip and the strike technique). The levels of impact force calculated for rigid bodies then have to be to put into relation with biomechanical loading thresholds of the stressed tissue (typically the bone of the skull vault). However, due to several unknown influencing factors, the physical components of such action as well as the biophysiological aspects can vary within a rather wide spectrum [8, 11]. In addition, the weight and mechanical stability of the steins are important factors when evaluating such events [14, 15]. Furthermore, individual anatomical characteristics of the head and its tissue specific biomechanical tolerance limits are most often not ascertainable in such incidents. The mechanical properties of the used beer stein, especially its state of use as well as the exact kinematics of its movement before impact are mostly not known. Therefore, laboratory tests performed in a way and manner, which are as close to a real incident as possible, are used as a database to support a reconstruction of events, based on comparative analytical analyses [8–11, 14, 15].

The patient at hand suffered from a rather small, but clearly identifiable, approximately 2-mm deep impression fracture of the skullcap. By means of a comparison with available literature, it appears likely that a force of at least 4-5kN affected the skull [11]. Taken into account the possibility of lower demolition thresholds of used beer steins [11, 15] and possible low individual biomechanical tolerance of the victim, an insignificantly lower force is possible. In order to reach such level of applied force, it can be assumed that the beer stein was hit with high intensity. The impact velocity may be expected in the range of 8–13 m/s [11].

If one compares the maximum possible force transmission in beer stein blasts to the head with the fracture tolerances of the skull bones [16–21], it can be stated that it is at least possible, regardless of the striking technique, to cause skull fractures. Thus, the occurrence of a skull fracture is plausible in this case. While skull fractures are not life threatening per se, they can be associated with possibly fatal complications (especially intracranial haemorrhages and secondary consequences such as inflammatory reactions). In the case at hand, the impact was demonstrably hard enough to cause a fracture of the skull vault. Consequently, the strike with a 1-l beer stein, in the way and manner it was applied in the discussed case, has to be assessed as a potentially life-threatening assault.

In this context, it has to be mentioned that a structural failure of one of the contacting objects (beer stein vs. skull) has an effect on the further increase of the impact force. Consequently, the victims in cases with beer stein breakup on the skull typically have superficial, soft tissue injuries.

## Limitations

There is a lack of information regarding the exact striking technique used, the state of the beer stain as well as the individual anatomical tissue thresholds of the victim. As this is not uncommon in physical assault cases, it is the task of the police and the forensic pathologist to try to fill in the blanks, where it is possible and to interpret to the best of their knowledge, where necessary information is not given.

## Conclusion

In the present case report, the victim suffered from a wound to the parietal region of the head as well as underlying soft tissue haemorrhages, swellings, and a skullcap impression fracture. This injury pattern is a remarkable case example of combined blunt and sharp-force trauma to the head and neck in which both, the skull as well as the striking weapon fractured.

Forensic biomechanical assessment was able to reconstruct the event as a single bottom-first, high-intensity blow to the head with a 1-l beer stein.

The action was assessed as a nonacute but potentially life-threatening assault.

The case was indicted as grievous bodily harm.
